# Establishing a Longitudinal Comparable Scale of Chinese Children's Cognitive Development through Calibrated Projection Linking

**DOI:** 10.3389/fpsyg.2018.00097

**Published:** 2018-02-22

**Authors:** Xiangzi Ouyang, Qiusi Zhang, Tao Xin, Fu Liu

**Affiliations:** ^1^Institute of Developmental Psychology, Beijing Normal University, Beijing, China; ^2^Department of English, Purdue University, West Lafayette, IN, United States; ^3^Collaborative Innovation Center of Assessment toward Basic Education Quality, Beijing Normal University, Beijing, China; ^4^Shenzhen Seaskyland Educational Evaluation Co. Ltd, Shenzhen, China

**Keywords:** children cognition, measurement invariance, longitudinal study, multidimensional IRT, calibrated projection

## Abstract

In the past decades, the longitudinal approach has been remarkably and increasingly used in the investigations of children's cognitive development. Recently, many researchers have started to realize the importance and necessity of examining measurement invariance for any further longitudinal analysis. However, there are few empirical studies demonstrating how to conduct further analysis when the assumption of measurement invariance of an instrument is violated. The primary purpose of this study is to explore how a newly-developed calibrated projection method can be applied to reduce the impact of lack of parameter invariance in a longitudinal study of preschool children's cognitive development. The sample consisted of 882 children from China who participated in two waves of the cognitive tests when they were 4 and 5 years old. Before this study was conducted, the IRT method was used to examine the measurement invariance of the instrument. The results showed that five items presented difficulty parameter drift and three items presented discrimination/slope parameter drift. In the study, the invariant items were treated as “common items” and calibrated projection linking was used to establish a comparable scale across two time points. Then the linking method was evaluated by three properties: grade-to-grade growth, grade-to-grade variability, and the separation of distributions. The results showed that the grade-to-grade growth across two waves was larger and exhibited a larger effect size; the grade-to-grade variability showed less scale shrinkage, which indicated a smaller measurement error; the separation of distributions showed a larger growth as well.

## Introduction

When the trend of children's cognitive development is assessed, the longitudinal approach is important because it facilitates the understanding of the dynamic processes of developmental change in children's cognition. As opposed to describing cognitive skills at different ages (Ornstein and Haden, 2001), longitudinal studies place an emphasis on developmental change and can elucidate developmental trajectories of skill acquisition (Grammer et al., 2013). For example, some longitudinal studies showed a systematic transition from relatively passive to more active remembering across elementary school years (e.g., Schneider and Sodian, 1997; Sodian and Schneider, 1999). The age-related trends revealed a picture of gradual development throughout childhood. In addition, the longitudinal method enables an examination of the mechanisms that may underlie the developmental changes as well as the skills associated with the changes over time. For example, Grammer et al. (2011) used the latent curve model to estimate the trajectories of children's strategy use and metamemory, which showed that the use of subsequent strategy is predictable by the metamemory at earlier time points.

Traditionally, the studies of children's cognitive development often relied on comparisons of manifest scale scores over time. For every child, the item scores of each scale would be averaged at each wave. The means were then compared using either paired-samples *t-*tests, when there were two measurement waves, or repeated measures ANOVA or other latent growth models, when more than two measurement waves were involved.

However, such a simple comparison of manifest scale scores over time may yield inaccurate results when the measurement of the underlying scale is not equivalent over time. That is because the manifest scale scores for the children's cognition scale depends not only on the latent true cognition score at each wave, but on the whole underlying measurement model (Steinmetz et al., 2009). As the children's cognitive ability develops fast in the preschool period, a unified instrument of a cognitive test is most likely inappropriate across different ages (e.g., some items are too hard or too easy for different ages). Therefore, the measurement invariance of the scale should always be ensured in a longitudinal comparison (Marsh and Grayson, 1994; Wu et al., 2010). Otherwise, it would be difficult to explain whether the changes in the manifest scale scores are due to the actual cognitive development (changes in the latent means) or merely the changes of the measurement (Vaillancourt et al., 2003). Thus, if the scale of the longitudinal measurement is not stable, conclusions derived from comparisons of manifest scale scores over time will be untrustworthy (Shadish et al., 2002).

### Measurement invariance

Measurement invariance is defined as the stable property of psychometric features of an instrument across different situations or time periods (Mellenbergh, 1989; Meredith and Millsap, 1992).

Establishing measurement invariance is a critical requirement for making inferences about treatment effects and changes in constructs over time. Ensuring that the structure of the measures remains stable over time can reduce measurement error and maximize the interpretability of the findings (Pitts et al., 1996). Therefore, longitudinal measurement invariance should be guaranteed before any further longitudinal analysis. Willoughby et al. (2012) investigated the longitudinal measurement invariance of Executive Function task battery before further longitudinal analysis, and found that two tasks exhibited partial measurement non-invariance, although the performance on the entire battery was stable over time.

Both the confirmatory factor analysis (CFA) method and the item response theory (IRT) method can be used to investigate measurement invariance. In CFA framework, a series of tests are required to investigate the measurement invariance, including tests for variance-covariance matrices, configural invariance, factor loadings invariance, intercept invariance, etc. (Schmitt and Kuljanin, 2008). Unlike the CFA approach, which often examines the measurement invariance at a test level, IRT is conducted at both the overall test level and item level. The examination of measurement invariance in the IRT framework can provide information on whether the discrimination/slope (*a* parameters) or difficulty (*b* parameters) of each item has changed across different time periods or situations, which is beneficial to the revision of items. Meade et al. (2005) compared the CFA and IRT methods in establishing measurement invariance. By utilizing a longitudinal assessment of job satisfaction as an example, they demonstrated that the differences in items' difficulty parameters over time could be effectively detected by IRT rather than CFA.

In many previous studies, researchers have attempted to examine measurement invariance before conducting longitudinal analysis and reported partial measurement invariance when some items showed drifted parameters (Willoughby et al., 2012; Hakulinen et al., 2014). For example, Meade et al. (2005) examined the measurement invariance of the instrument of job satisfaction using IRT, and the results indicated that three items functioned differently at Time 1 (T1) and Time 2 (T2). However, there has been a lack of discussions in literature about the solution to such a problem in longitudinal studies.

The solution proposed in the present study is “calibrated projection linking.” This is a newly-developed method, which was previously used in linking parallel tests (Thissen et al., 2011; Cai, 2015). Calibrated projection involves a two-tier IRT model (Cai, 2010) to link two measures, which is distinct from the conventional calibration that requires the two measures to be of the same construct. It, therefore, allows the lack of measurement invariance of the instrument. In linking parallel tests without common items, the nearly identical item pairs in the two instruments were set to be common items to link scores on the PedsQL Symptoms Scale to the IRT metric of the PROMIS pediatric asthma impact scale (PAIS) (Thissen et al., 2011).

The present study aims to explore the applications of calibrated projection to establish a longitudinal comparable scale of a 4- to 5-year-old children cognitive development test, of which some items lacked measurement invariance. The cognitive ability growth of the children from ages 4 to 5 is first described. The procedure of applying calibrated projection linking in the longitudinal studies is then illustrated with an example of a cognitive development test. Last, the performance of this method is presented and discussed. Overall, this study can be of interest to both substantive and methodological researchers.

## Methods

### Measures

The instrument in this study is part of a series of instruments in a project on the Chinese national 3- to 6-year-old children's learning and development. The instrument was designed with heavy reference to the Chinese version of the Binet test and WISC-IV and was then refined after 30 psychologists expertized in the children's cognitive development were interviewed. The refined cognitive test consists of nine items: comparing quantity, orientation, addition and subtraction, jigsaw, classification, sorting, patterning, measurement, and fetching objects. Each item consists of four tasks at different levels, ranging from easy to difficult. The children started their test at different levels according to their ages. For instance, the 4-year-olds started at level 1, and 5-year-olds level 2. Only when they accomplished one task could they move on to the next level. At last, their performances were scored according to how many tasks they completed. Each task was worth 1 point, so the score of each item ranged from 0 to 4. Every child was tested by an experimenter who received professional training.

### Samples

The data of this study were derived from part of the project mentioned above, which was conducted by UNICEF and Ministry of Education in China. The sample consists of 882 children from different provinces across China, including Inner Mongolia, Sichuan, Heilongjiang, Hebei, Jiangsu, and Fujian. Of all the 882 children, 422 (48%) were male and 460 (52%) were female. In addition, 460 (52%) were from urban areas and 422(48%) were from rural areas. All of the procedures conducted in the study were approved by the Institutional Review Board (IRB) and the participants' parents.

### Analysis

In the study, the two-tier IRT model (Cai, 2010) was used to link the cognitive tests across two time points. The two-tier model describes the probability of each item response as a function of a set of item parameters and the latent variables measured by the scale. The rationale of adopting the two-tier model rests on the following facts. Firstly, from a substantive view, young children tend to develop an understanding of mathematical concepts, which are reflected by their informal ideas of more and less, taking away, shape, size, location, time, pattern, and position (Baroody et al., 2006; Clements and Sarama, 2009; Lee et al., 2009). The two-tier model can model both the general factor (mathematic ability) and specific dimensions (e.g., “classification”) at the same time. Secondly, from a methodological view, the two-tier model is suitable for investigating a longitudinal study, since it takes into account the time effects of the general dimension, which represents the mathematical abilities at ages 4 and 5 (θ_1_ and θ_2_ in **Figure 2**). The two-tier model for graded response (Samejima, 1969, 1997; Cai, 2010) is denoted as

$$\begin{array}{lll} P_{asjk} & = & P_{asj,\text{k}}^{*}-P_{asj,\text{k}+1}^{*}\\ P_{asj,0}^{*} & = & 1\\ P_{asj,\text{k}+1}^{*} & = & 0\\ P_{asj,\text{k}}^{*} & = & \frac{1}{1+\exp\left\{-\left[\beta_{jk}+{a_{a}}^{\prime }\theta_{a}+a_{s}\zeta_{s}\right]\right\}}\\ \text{ for k}=1,2,3,4,5\ldots \left(\text{Response categories}\right), \end{array}$$

where subscript *k* represents the response category; *j* represents the items; subscript *a* represents the general dimension; subscript *s* represents specific dimensions; **a**_*j*_ is the vector of slope parameters; **θ**_*j*_ is the vector of abilities at different time points; β_*jk*_is the intercept parameter; ***a***_*a*_ is the slope parameter of general dimension; *a*_s_ is the slope parameter of specific dimension; ζ_s_ is the ability of specific dimension.

### Calibrated projection

**Figure 1:**
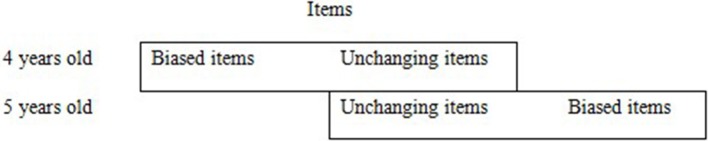
Linking design of 4–5 years old children cognitive test.

**Figure 2:**
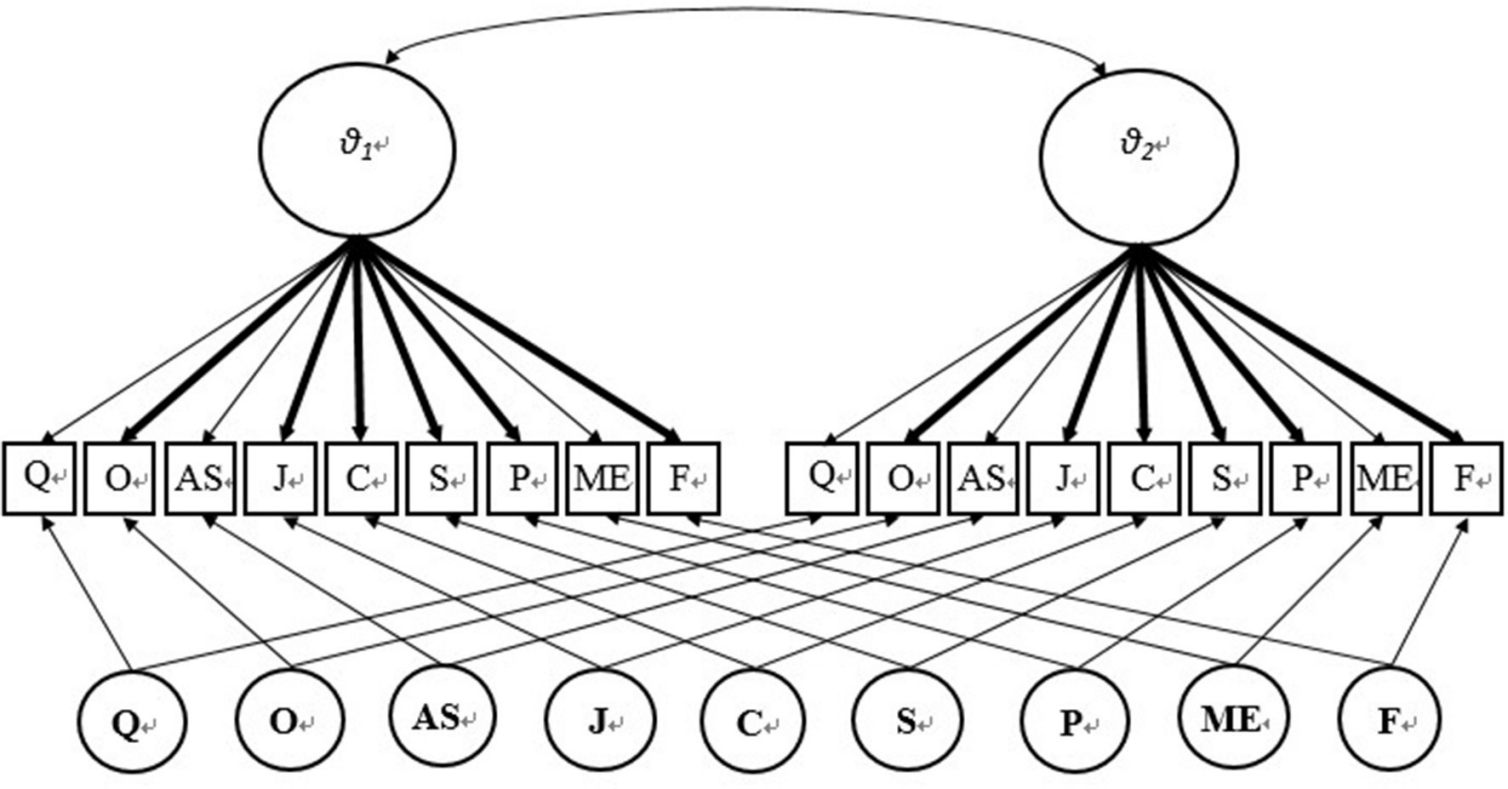
Two-tier model for linking. Q, Comparing quantity; O, Orientation; AS, Addition and subtraction; J, Jigsaw; C, Classification; S, Sorting; P, Patterning; ME, Measurement; F, Fetching.

Calibrated projection is a new statistical procedure that exploits the two-tier IRT model to link two measures (Thissen et al., 2011). With calibrated projection, the item responses from two time points were fitted to the model presented above: θ_1_ denotes the underlying cognitive ability of the 4-year-olds, and θ_2_ denotes the cognitive ability of the 5-year-olds. Prior to this study, Ouyang et al. (2016) conducted a study that examined measurement invariance using the IRT model on the same instrument. The results of the study indicated that three items exhibited *a* parameter drift: “comparing quantity” ($\Delta \chi_{1}^{2}=6.87,p<0.01$), “addition and subtraction” ($\Delta \chi_{1}^{2}=14.17,p<0.01$), and “measurement” ($\Delta \chi_{1}^{2}=6.86,p<0.01$). Five items exhibited category intercept *(d)* parameter drift: “comparing quantity” ($\Delta \chi_{4}^{2}=50.71,p<0.01$), “addition and subtraction” ($\Delta \chi_{4}^{2}=28.67,p<0.01$), “orientation” ($\Delta \chi_{4}^{2}=140.34,p<0.01$), “jigsaw” ($\Delta \chi_{4}^{2}=27.65,p<0.01$), and “classification” ($\Delta \chi_{4}^{2}=26.06,p<0.01$). In order to place the items at two time points on the same scale, this study treated the invariant items as “common items,” which is shown in Figures 1, 2, and then the *a* parameters of each common item at T1 were set equal to the *a* parameters for its counterpart at T2. The common items for linking *a* parameters are “Orientation,” “Jigsaw,” “Classification,” “Sorting,” “Patterning,” and “Fetching Objects,” which are the bold lines in Figure 2. The category intercept parameters of each of the common items were also consistently set equal across the two time points. The common items for linking β parameters are: “Sorting,” “Patterning,” “Measurement,” and “Fetching Objects.” Then the IRT scale ability scores were estimated at two time points, and then transformed to T-scores for the convenience of comparison.

### Evaluation

In the study, as calibrated projection was applied in real samples rather than simulated samples, very few properties could be employed to evaluate the performance of the proposed approach. Therefore, the properties often applied in evaluating vertical scaling in longitudinal studies were adopted, which are grade-to-grade growth, grade-to-grade variability, and the separation of grade distributions (Kolen and Brennan, 2004; Kim, 2007). The method without calibrated projection was used as the baseline in the study. Thus, the performance of calibrated projection was evaluated by comparing the properties with the ones of the baseline method.

Grade-to-grade growth is defined as “the change from one grade to the next over the content taught in a particular grade” (Kolen and Brennan, 2004, p. 377). The indicator of grade-to-grade growth is the mean difference between consecutive grades. The mean estimates are expected to increase with age regardless of content areas or the proficiency estimators used (Kim, 2007). By examining the mean value at each age, questions like the following can be answered: how much do students grow, on average, from 1 year to the next? Are growth patterns different at different ages?

Grade-to-grade variability refers to the pattern of within-grade variability at different ages. Hoover (1984) argued that within-grade variability should increase with age because in young ages low-achieving students are expected to grow at a slower rate than high-achieving students. The indicator of grade-to-grade variability is the difference among the standard deviations (SDs) of each age on their own scale. Any dramatic change over grades, say 10 times larger or smaller, would indicate that the scale might not be functioning well.

The separation of grade distributions is the degree of overlap between scale score distributions of consecutive grades. One index of the separation of grade distributions is the horizontal distances between the distributions of consecutive grades (Holland, 2002), which is based upon the difference between the two distributions at selected percentile points (Holland, 2002; Kim, 2007). To compute horizontal distances, certain percentile points of the score distributions must be selected. If *p* denotes a percentile point, then the p-percentile, *X(p)*, of the cumulative distribution function (CDF), *F*, is defined as

(1) $$\begin{array}{r} p=F\left[X\left(p\right)\right]\;or\;X\left(p\right)=F^{-1}\left(p\right), \end{array}$$

*X*(*p*) is usually referred to as “the *p*th percentile” of *F*, and *F*^−1^(*p*) denotes the inverse function of *F*. Likewise, the percentiles of another CDF of G can be denoted by *Y(p)*. Then, using Equation (1),

(2) $$\begin{array}{r} p=G\left[X\left(p\right)\right]\;or\;Y\left(p\right)=G^{-1}\left(p\right) \end{array}$$

Then, the horizontal distance between two distributions of *F* and *G, HD(p)*, can be defined as:

$$\begin{array}{l} HD\left(p\right)=Y\left(p\right)-X\left(p\right) \end{array}$$

*HD*(*p*)represents the difference between the *p*th percentiles of the two distributions. For example, for the distribution of 4-year-old children, *F*, the percentile rank of a θ of 1, is 50. For the distribution of 5-year-old children, G, the percentile rank of a θ of 1.3, is 50. By Equations (1–2), the horizontal distance between the two distributions of *F* and *G* at the 50th percentile is 0.3.

Horizontal distances were computed to examine the gaps at selected locations throughout the entire distributions: 5th, 10th, 25th, 50th, 75th, 90th, and 95th. In the present study, horizontal distances of the scale with calibration projection applied were compared with the ones of the baseline method. Any dramatic change over grades or percentile points, say 10 times larger or smaller, would indicate that the scale might not be functioning well (Kim, 2007).

Calibrated projection process was conducted using IRTPRO 2.1, and the outputs from IRTPRO 2.1 were then analyzed using SPSS20.

## Results

### Descriptive statistics

Table 1 shows the reliability of the children's cognitive test at two time points. In psychological tests, alpha coefficient 0.7 is the cut-off value for being acceptable (Santos, 1999). As our cognitive tests only included 9 items, the reliability of the test is acceptable.

**Table 1:** Cronbach's alpha coefficient.

**Time points**	**Cronbach's α coefficient**
Wave 1	0.70
Wave 2	0.72

### Linking the children's cognitive longitudinal test

The present study used the two-tier IRT model (Cai, 2010) to link the test across two waves. Table 2 shows the item parameters of the cognitive test at two time points. The invariant item parameters across two time periods were bold in the table. The 3rd−7th columns show the slope (*a*) parameters on the general dimension representing the time effects of the mathematics test and the category intercept (β) parameters that were freely estimated in the two-tier model. In the 9th−13th columns are the item parameters obtained after the common items were constrained to be equal. The 8th and 14th columns show the item slope (*s*) parameters on specific dimensions of mathematics, such as “comparing quantity,” “addition and subtraction,” and “orientation,” etc., which were fixed because the contents of the nine items did not change in two waves. The correlation between cognitive abilities of the 4- and 5-year-olds is 0.86.

**Table 2:** Item parameters with and without linking.

**Items**	**Time points**	**Without linking**						**With linking**					
		***a***	***β_1_***	***β_2_***	***β_3_***	***β_4_***	***s***	***a***	***β_1_***	***β_2_***	***β_3_***	***β_4_***	***s***
Q	T1	0.62	2.09	−0.45	−1.26	−2.14	0.49	0.61	2.16	−0.37	−1.19	−2.07	0.50
	T2	1.06	2.16	−0.55	−1.23	−1.51		0.91	1.54	−1.17	−1.86	−2.14	
O	T1	1.15	2.57	−0.13	−2.90	−6.99	0.65	**1.03**	2.58	−0.04	−2.75	−6.80	0.64
	T2	1.10	4.38	1.65	−0.82	−2.71		**1.03**	3.74	0.96	−1.55	−3.47	
AS	T1	1.20	0.18	−0.13	−1.31	−3.04	0.49	1.19	0.34	0.02	−1.16	−2.88	0.49
	T2	0.87	3.43	2.83	−0.20	−2.29		0.74	2.93	2.33	−0.71	−2.79	
J	T1	1.02	1.41	0.73	−2.79	−4.54	0.92	**1.10**	1.61	0.92	−2.66	−4.42	0.93
	T2	1.38	2.96	2.53	−1.94	−2.99		**1.10**	2.17	1.75	−2.64	−3.68	
C	T1	1.03	1.77	−0.97	−4.36	−5.88	0.72	**1.00**	1.88	−0.85	−4.23	−5.74	0.72
	T2	1.16	2.33	0.30	−2.40	−3.71		**1.00**	1.65	−0.39	−3.10	−4.42	
C	T1	1.60	0.74	−0.49	−1.49	−2.47	0.71	**1.66**	**0.85**	**−0.32**	**−1.24**	**−2.23**	0.71
	T2	2.26	1.87	0.76	−0.13	−1.19		**1.66**	**−0.85**	**−0.32**	**−1.24**	**−2.23**	
P	T1	1.34	2.89	0.16	−0.64	−1.40	0.61	**1.23**	**3.00**	**0.31**	**−0.58**	**−1.32**	0.60
	T2	1.32	4.01	1.20	0.23	−0.48		**1.23**	**3.00**	**0.31**	**−0.58**	**−1.32**	
ME	T1	0.83	0.57	−1.08	−2.35	−3.40	0.77	0.85	**0.68**	**−0.90**	**−2.04**	**−3.20**	0.76
	T2	1.36	1.49	0.01	−1.05	−2.25		1.25	**0.68**	**−0.90**	**−2.04**	**−3.20**	
F	T1	1.17	4.15	0.96	−2.26	−3.74	1.54	**1.29**	**4.35**	**1.28**	**−2.02**	**−3.54**	1.52
	T2	1.44	4.98	2.29	−1.09	−2.63		**1.29**	**4.35**	**1.28**	**−2.02**	**−3.54**	

*Q, Comparing quantity; O, Orientation; AS, Addition and subtraction; J, Jigsaw; C, Classification; S, Sorting; P, Patterning; ME, Measurement; F, Fetching Objects*.

The slope parameters represent the discrimination of the items in the IRT framework, and 0.64 or greater is considered as moderate or high discrimination (Baker, 2001). Thus, all items, except “Comparing quantity,” were highly discriminating. β parameter represents the category intercept parameter, which is opposite to difficulty parameter. The higher the β parameter is, the easier the task level. For example, in Table 2 “Measurement” is more difficult than “Patterning” at all task levels. By comparing the category intercepts of “addition and subtraction,” it can be seen that β_1_ and β_2_ drifted severely across two waves (0.34, 0.22 at the first wave and 2.93, 2.33 at the second wave), which indicates that the first and second task levels may have been too easy for 5-year-old children. Furthermore, the slope parameters can be compared between general and specific dimensions. For example, the slope parameters of “addition and subtraction” on the general dimension are 1.20 and 0.87, and the one on the specific dimension is 0.49. This indicates that “addition and subtraction” explained more variability of the entire mathematics test. In contrast, the slope parameters of “fetching objects” on the general dimension are 1.17 and 1.44, and the one on the specific dimension is 1.54. This indicates that this item is highly related to both specific and general dimensions.

### Evaluation

After the calibrated scale was established, the ability parameters were estimated, and then transformed to T-score for convenience, which is

$$\begin{array}{l} T=10\;\theta +50. \end{array}$$

**Figure 3:**
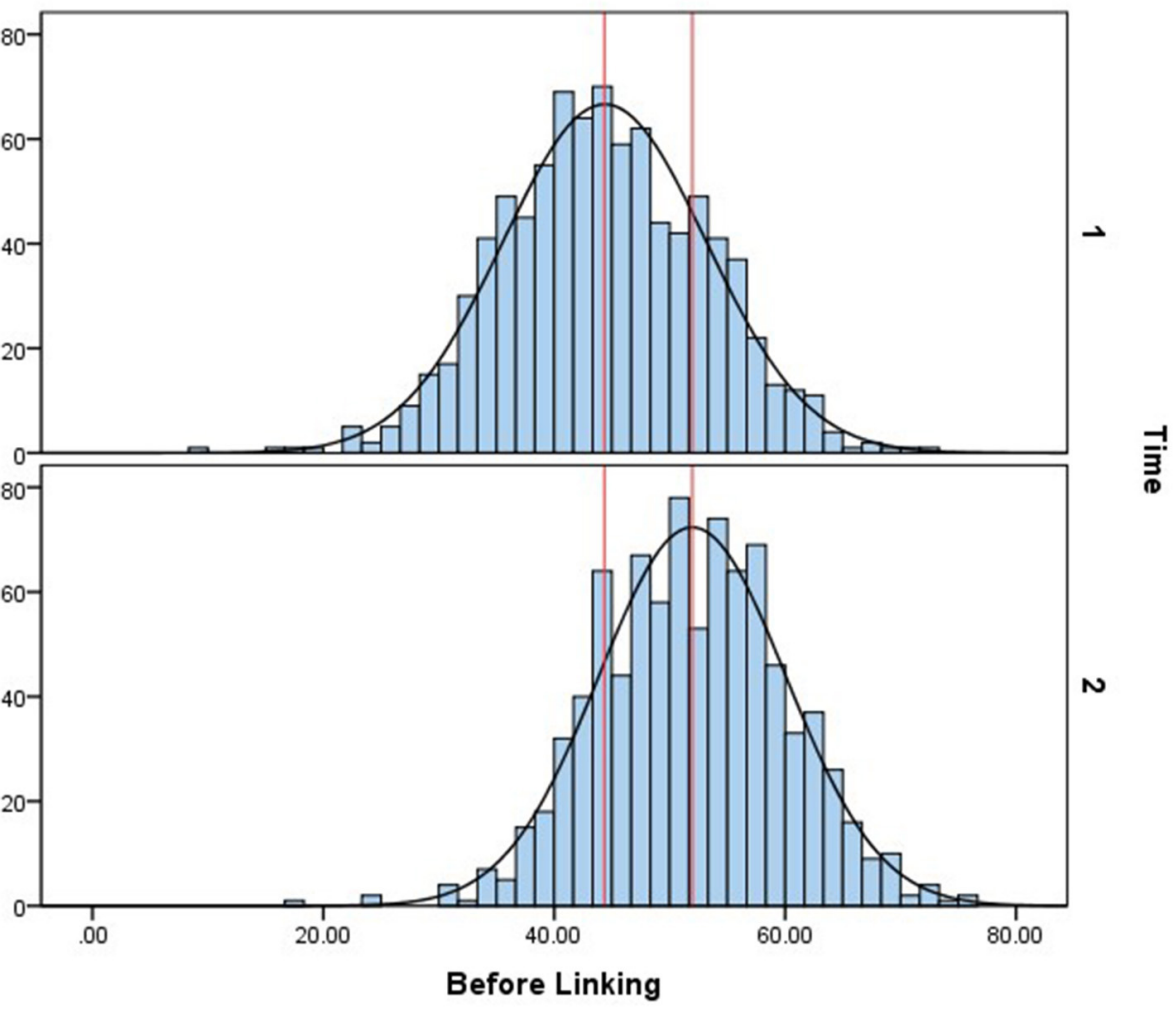
Ability distribution without calibrated projection.

**Figure 4:**
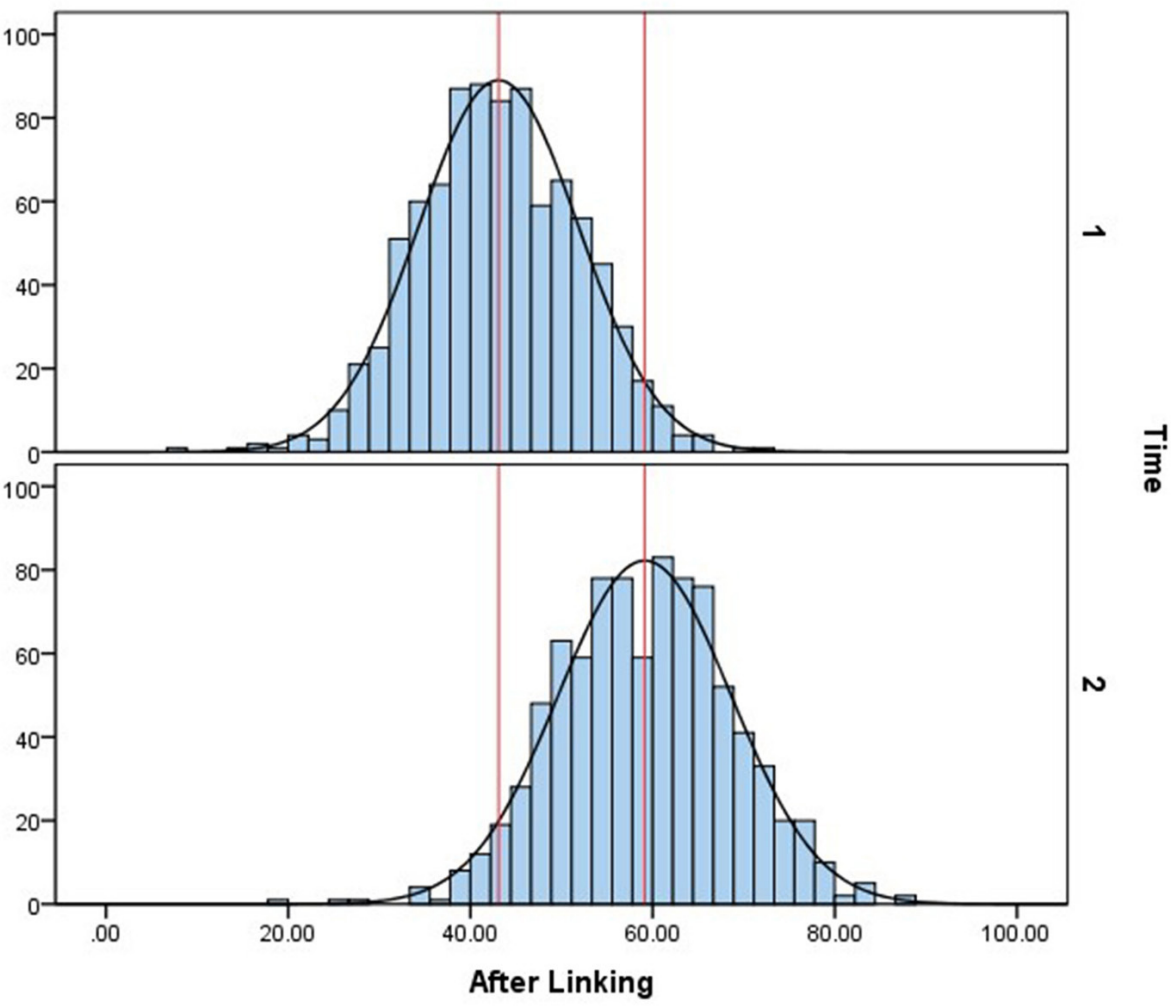
Ability distribution after calibrated projection.

The ability distributions without linking and with linking are both shown below. Figure 3 is the histogram and normalized ability distribution of the baseline method, which means all slope parameters on the general dimension of the nine items in the two-tier model were freely estimated. In this figure, the upper graph shows the cognitive ability distribution of the 4-year-old children, and the lower graph shows that of the 5-year-old children. The red lines in both graphs denote the means of the two distributions. The mean of 4-year-old children's cognitive ability is 44.36, and that of the 5-year-olds is 51.96. Figure 4 shows the normalized cognitive ability distributions of 4- to 5-year-old children after calibrated projection was applied. The mean of the 4-year-olds is 43.08, and that of the 5-year-olds is 59.12. By comparing Figures 3, 4, it can be seen that with calibrated projection, the ability distributions presented a larger growth across two waves, which is aligned with the findings of some previous studies that the preschool is a key period in which children's cognitive ability grows rapidly (Chang, 2009).

### Grade-to-grade growth

**Figure 5:**
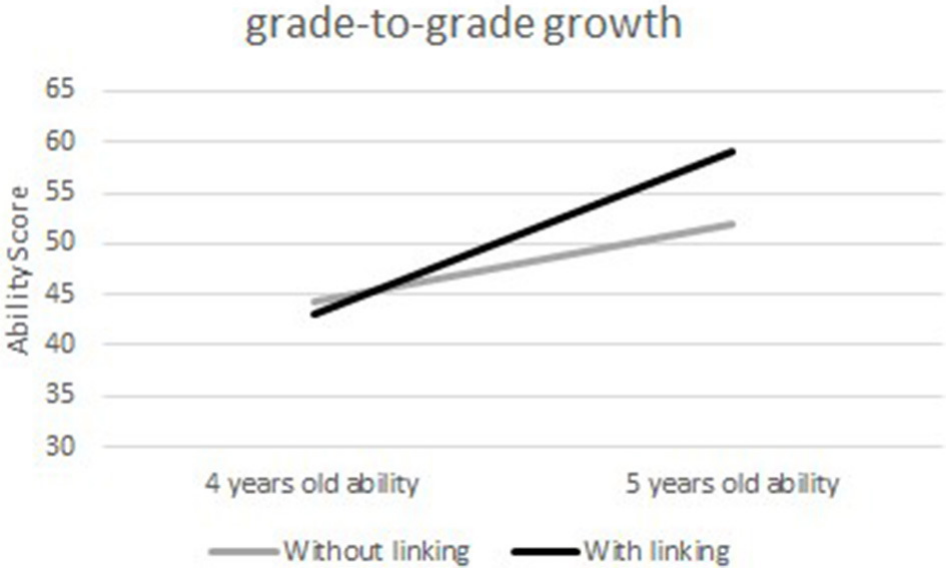
Comparison between 4 and 5 years old ability means without and with linking.

In the present study, grade-to-grade growth means the average ability growth between 4- and 5-year-old children. In Figure 5, the average ability score is increased by 7.59 without linking and 16.04 with linking. This difference indicates that, with calibrated projection, there was a larger growth in the cognitive ability of children from ages 4 to 5.

Furthermore, by paired sample *T*-test, the significance and effect size of children's ability growth with and without linking were compared. Table 3 shows that regardless of whether the linking was applied or not, the ability growths of children from ages 4 to 5 are both significant. However, the effect size with linking is almost twice as large as the one of the baseline method.

**Table 3:** Paired sample *T*-test for score difference between 4- and 5-year old children without and with linking.

	**Mean**	***SD***	**SE**	***t***	**Cohen's d**
Without linking	−7.60	2.82	0.09	−80.12^***^	0.10
With linking	−16.04	3.07	0.10	−155.03^***^	0.19

*** *p < 0.001*.

### Grade-to-grade variability

**Figure 6:**
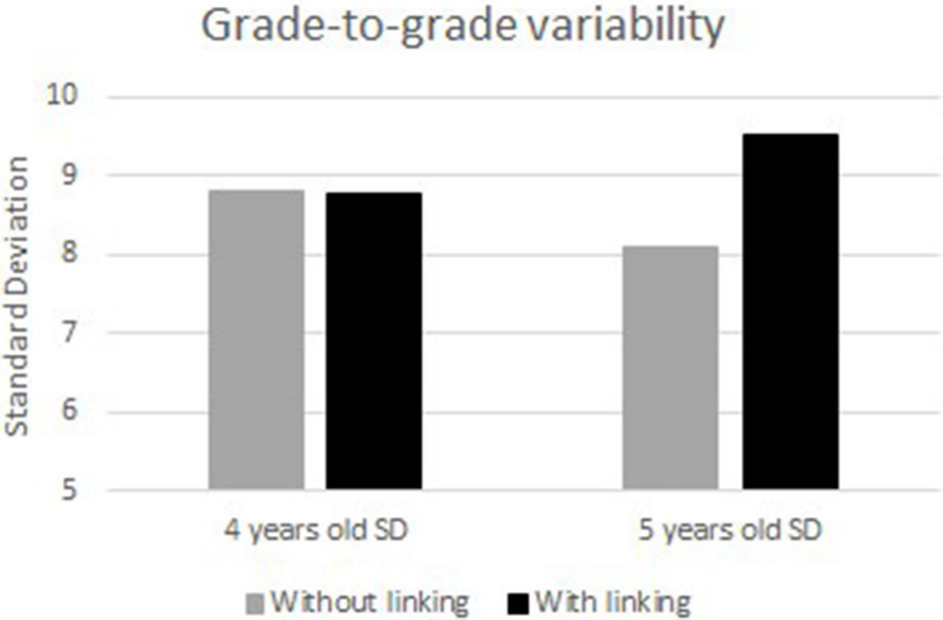
Comparison between 4 and 5 years old ability standard deviation without and with linking.

Figure 6 shows the differences in the standard deviations between the two scales obtained with and without linking. With linking, the SDs at two time points are 8.79 and 9.51, respectively. Without linking, however, the SDs at two time points are 8.80 and 8.10, respectively, showing a “scale shrinkage” problem (Hoover, 1984, 1988). It implies a decrease in variability of the score with age.

### Separation of grade distributions

The separation of grade distributions is mainly demonstrated by horizontal distance (HD, Holland, 2002). To examine gaps between distributions of consecutive grades, the HDs were computed at the following selected percentile points: 5th, 10th, 25th, 50th, 75th, 90th, and 95th, and then averaged. As is shown in Table 4, the average HD is 7.56 without linking and 16.04 with linking. This result indicates that the difference of ability distributions between two time points is larger after calibrated projection was applied, which is consistent with the result of grade-to-grade growth.

**Table 4:** Comparison between 4 and 5 years old ability average HD without and with linking.

**Percentile HD**	**Without linking**	**With linking**
5th	8.78	15.12
10th	8.38	14.96
25th	8.01	15.37
50th	7.90	16.20
75th	6.67	16.12
90th	6.69	16.94
95th	6.51	17.56
Average HD	7.56	16.04

## Discussion

In longitudinal studies, measurement invariance is a significant property that needs to be established before any further analysis is conducted. In the field of children's cognitive development, as the children's cognitive abilities grow very fast during the childhood, the instrument can be aptly drifted across different ages. The previous study of examining the longitudinal measurement invariance (Ouyang et al., 2016) showed that among nine items, 3 *a* parameters and 5 category intercept (β) parameters presented a drift across two waves, although the construct of the test over two waves remained stable by reference to the high correlation of 0.86. As so many item parameters were drifted over time, the reliability or predictive validity of the test could have been compromised (e.g., Alvares and Hulin, 1972; Henry and Hulin, 1987). In order to achieve a more accurate measurement of children's cognitive developing trajectory from 4- to 5-year-old, calibrated projection was applied to establish a comparable scale in this longitudinal test.

Calibrated projection was mostly applied to link parallel tests in previous studies (e.g., Thissen et al., 2011; Monroe et al., 2014). The present study extended the method to reduce the impact of lack of measurement invariance in longitudinal tests. Calibrated projection is based on the two-tier IRT model (Cai, 2010), of which each item loads on both general dimensions and a specific dimension. In the previous studies of linking parallel tests, the item parameters that loaded on the specific dimension representing the same content of the items across two tests were set equal so as to play roles of “common items.” However, in the present longitudinal study the common item parameters that load on the general dimension representing the time effect were set equal during the process of estimation. Furthermore, the category intercept (β) parameters of common items were also set to be equal to reduce the item difficulty parameters drift. The IRT method of examining measurement invariance can provide more information about the performance of different items. For example, Table 2 shows that the first two tasks of “addition and subtraction” may be too easier for 5 years old children, which need revision in future.

In order to compare the ability scales established by the proposed method and the baseline method, three evaluation criteria that have been used in vertical scaling were adopted in the study. They are grade-to-grade growth, grade-to-grade variability, and separation of grade distributions (Kim, 2007). These criteria were represented by mean difference, standard deviation (SD), and average horizontal distance (HD), respectively (Hoover, 1984; Camilli, 1988; Kim, 2007).

First of all, with calibrated projection, the mean difference shows a larger growth of children's cognitive ability from ages 4 to 5. Furthermore, the statistical test shows that the mean differences are significant in both cases, but the effect size was larger with linking. The growth pattern with calibration project applied in this study provides strong supports for many previous studies about the rapid development of children's cognition from ages 4 to 5 (Chang, 2009; Zhao, 2009). For example, Yang (2009) suggested that Chinese children younger than 4 can only accomplish the task of sorting 4 items, while children at 5 can accomplish 10 items.

Secondly, the result of the grade-to-grade variability shows that within-grade SD with calibrated projection applied increases with age, which supports Hoover's (1984) study. Hoover explained the reason of the result, based on the expectation of a slower growth rate of low-achieving students than high-achieving students at young ages. This growth pattern of mathematic ability across preschool years is also supported by other studies (Bast and Reitsma, 1997; Aunola et al., 2002, 2004). In addition, the grade-to-grade variability shows a decrease with age with the baseline method, which indicates “scale shrinkage” (Kim, 2007). According to Hoover (1984, 1988), scale shrinkage is not very common in real data. Camilli (1988) indicated that scale shrinkage may be caused by systematic estimation error or measurement error, drawing on the findings of some simulation studies that if the variability of item parameters is set to be different, then more scale shrinkage problems would occur. Therefore, scale shrinkage problem that occurred in the cognitive ability distributions with the baseline method suggests that there might exist some systematic estimation error.

Thirdly, the separation of grade distributions represents the difference between the ability distributions in two waves. In the present study, the comparison of average HD indicates that with calibrated projection, the difference between ability distributions from age 4 to 5 is larger, which is similar to the result of grade-to-grade growth. The consistency between the HD results and the results of grade-to-grade growth is also indicated in some previous studies (Kim, 2007).

## Limitations and conclusion

Despite the strengths of this study, there also exist a few limitations. Firstly, the sample only covered 4- to 5-year-old children, and the 1-year range was limited for further analysis. In some vertical scaling studies, the age range is often 3–4 years or more, which would yield more information about the scaling method by comparing evaluation properties across every 2 consecutive years. Thus, in future, the age range should be extended to 3–6 years old, the whole preschool stage for Chinese children, so as to investigate whether calibrated projection will performance in the same way when used to evaluate the growth across other consecutive ages. Secondly, as this study was conducted with a real sample, it constrained the use of the evaluation criteria. Because there is no standard value or true value for the grade-to-grade growth, variability, and the separation of grade distributions for comparisons. The evaluation in this study was mostly based on the results of previous studies about their performances in different situations. In the future, more simulation studies are needed to evaluate the performance of calibrated projection, so that the results can be compared with other linking or vertical scaling methods in longitudinal studies.

Despite these limitations, the current study demonstrates ways of applying calibrated projection method to link longitudinal tests when there occur item parameter drifts in the instrument across different waves. This is critical, as changes in the psychometric properties of a test over time could sacrifice its reliability or predictive validity (e.g., Alvares and Hulin, 1972; Henry and Hulin, 1987). Furthermore, the results of this study indicated that with linking, grade-to-grade growth and its effect size are larger. The result of grade-to-grade variability after linking is aligned with the result of the study of Hoover (1984) and shows less scale shrinkage, which indicates a smaller measurement error. Moreover, the conspicuous separation of grade distributions supports the result of grade-to-grade growth. In summary, comparisons of the three properties showed a possible consequence of ignoring the measurement invariance in a longitudinal analysis as well as the performance of calibrated projection from a practical view.

## Ethics statement

The study was approved by the Institutional Review Board (IRB) of Beijing Normal University. All the parents of participants provided written informed consent.

## Author contributions

XO wrote the first draft of the manuscript and assisted study design, and data analyses. QZ revised it critically for important intellectual content. TX was the principal investigator of the study and the data provider. All of the authors participated in the final approval of the version to be published and agreed to be accountable for all aspects of the work.

### Conflict of interest statement

The authors declare that the research was conducted in the absence of any commercial or financial relationships that could be construed as a potential conflict of interest.
